# Multimodal Brain Growth Patterns: Insights from Canonical Correlation Analysis and Deep Canonical Correlation Analysis with Auto-Encoder

**DOI:** 10.3390/info16030160

**Published:** 2025-02-20

**Authors:** Ram Sapkota, Bishal Thapaliya, Bhaskar Ray, Pranav Suresh, Jingyu Liu

**Affiliations:** Translational Research in Neuroimaging and Data Science (TReNDS) Center, Georgia State University, Atlanta, GA 30303, USA;

**Keywords:** multimodal, brain development, CCA, DCCAE

## Abstract

Today’s advancements in neuroimaging have been pivotal in enhancing our understanding of brain development and function using various MRI techniques. This study utilizes images from T1-weighted imaging and diffusion-weighted imaging to identify gray matter and white matter coherent growth patterns within 2 years from 9–10-year-old participants in the Adolescent Brain Cognitive Development (ABCD) Study. The motivation behind this investigation lies in the need to comprehend the intricate processes of brain development during adolescence, a critical period characterized by significant cognitive maturation and behavioral change. While traditional methods like canonical correlation analysis (CCA) capture the linear interactions of brain regions, a deep canonical correlation analysis with an autoencoder (DCCAE) nonlinearly extracts brain patterns. The study involves a comparative analysis of changes in gray and white matter over two years, exploring their interrelation based on correlation scores, extracting significant features using both CCA and DCCAE methodologies, and finding an association between the extracted features with cognition and the Child Behavior Checklist. The results show that both CCA and DCCAE components identified similar brain regions associated with cognition and behavior, indicating that brain growth patterns over this two-year period are linear. The variance explained by CCA and DCCAE components for cognition and behavior suggests that brain growth patterns better account for cognitive maturation compared to behavioral changes. This research advances our understanding of neuroimaging analysis and provides valuable insights into the nuanced dynamics of brain development during adolescence.

## 1. Introduction

Over the past decades, advancements in neuroimaging have played a crucial role in understanding brain development [1,2] contributing significantly to biomedical research [3,4]. Numerous studies have utilized traditional machine learning [5–7] and deep learning [8–12] to investigate various aspects of brain organization, including its anatomy, functional dynamics, and network connectivity. Various MRI techniques, including T1-weighted imaging, diffusion-weighted imaging, and task-based and resting-state functional MRI, along with genetic data, are increasingly employed to study brain structure and function more effectively [13–17]. Multimodal neuroimaging analyses, which combine data from two or more neuroimaging modalities, leverage complementary information and overcome the limitations of each [18]. This approach of multimodal fusion has yielded more reliable results [3,4,19], providing comprehensive insight into the understanding of brain and cognition, as well as abnormal brain function and mental disorders [4,20]. Recent studies also suggest that neural interfaces, which establish a direct link between the brain and external devices, can present new opportunities in the future for integrating multi-modalities to advance brain–computer communication [21].

Various approaches, such as parallel independent component analysis, canonical correlation analysis (CCA), and deep learning-based fusion, have been explored for integrating multimodal data, as has been extensively reviewed in [20,22–24]. This study specifically focuses on CCA-based methodologies due to their straightforward implementation and widespread use. CCA facilitates a linear transformation of data, and it reveals maximally linearly correlated hidden patterns [25]. An extension of CCA known as deep canonical correlation analysis (DCCA) leverages deep neural networks to capture the non-linear hidden features of each data modality and then maximizes the relationship between two sets of hidden features, resulting in highly correlated representations across data modalities [26,27].

This study examined Adolescent Brain Cognitive Development (ABCD) data. The ABCD Study includes a longitudinal cohort consisting of youth aged 9–10 at the baseline from 21 different sites across the US, and it is the largest long-term study that followed youths for 10 years with annual lab-based assessments and bi-annual imaging acquisitions [28]. It is designed to examine the interplay between biological, behavioral, and environmental factors on brain development and health outcomes in children and adolescents [29]. ABCD studies leverage the measurement of brain structure and function relevant to adolescent development and addiction, providing evidence for the feasibility and age-appropriateness of the procedures and the generalizability of findings [30]. Also, the study of neurocognitive development is crucial for distinguishing premorbid vulnerabilities from the consequences of behaviors like substance use [31].

We investigated the structural changes within two years of brain development between 9–10-year-olds and 11–12-year-olds using images of gray matter and white matter from the ABCD Study. Our hypothesis posits that gray matter and white matter grow coherently during this period, implying a significant relationship between changes in gray matter density and white matter integrity. Such brain-structural changes underline the observed cognitive maturation and behavioral changes outwardly. While CCA captures the linear interactions of brain regions, we recognize the potential contribution of non-linear interactions across brain regions. To address this, we implemented two approaches: CCA and an extension of DCCA, DCCA with an autoencoder (DCCAE), which incorporates both the reconstruction objective from an autoencoder and a standard CCA correlation objective [32]. Gray matter and white matter growth patterns extracted from both approaches were compared and contrasted. Furthermore, we explored their associations with cognitive and behavioral changes. The contribution of this study is twofold. This study aimed to understand the growth of GM and FA during the two years of critical brain development and their significant changes underlying cognitive and behavior changes, as well as to determine whether the brain growth pattern across regions is linear or non-linear.

## 2. Materials and Methods

### 2.1. Participants and Data

We employed baseline data (9–10 years) and two-year follow-up (11–12 years) data from two different types of imaging modalities: gray matter density (GM) images from T1 weighted structural MRI and white matter fractional anisotropy (FA) images from diffusion MRI. Following a quality check, the gray matter images consist of 11,573 baseline participants and 3947 two-year follow-up participants. Similarly, the white matter images consist of 10,267 baseline participants and 3745 two-year participants. The number of common participants with the baseline and follow-up of gray matter and white matter is 3302.

In this study, the cognitive measures we used are fluid intelligence (a person’s ability to handle new and unfamiliar situations and how quickly), crystallized intelligence (a person’s ability to apply knowledge previously gained through education and experience), and the total composite (which encompasses both fluid and crystallized elements). Behavioral assessments are derived from the Child Behavior Checklist (CBCL), a tool used to identify behavioral and emotional issues in children and adolescents, which is made up of eight syndrome scales: anxious/depressed, depressed, somatic complaints, social problems, thought problems, attention problems, rule-breaking behavior, and aggressive behavior. Two-year changes in the cognitive and behavioral measures were computed via the direct subtraction of the baseline from the year-two follow-up data and utilized for association analyses with brain-structural growth patterns extracted via both CCA and DCCAE approaches, as is explained later on.

### 2.2. Gray Matter Image Processing

T1-weighted MRI images were segmented into GM maps and normalized into Montreal Neuroimaging Institute (MNI) space using the Statistical Parametric Mapping 12 (SPM12) software toolbox. Then, the gray matter density maps were smoothed via a Gaussian Kernel (6 mm^3^), resliced to 1.5 mm^3^ voxels. Further quality control was conducted to retain GM maps with a correlation greater than 0.9 with the mean GM map across all the individuals.

### 2.3. White Matter Image Processing

Diffusion MRI images were preprocessed using FSL (v6.0.1) for distortion correction, eddy current correction, and motion correction, and then they were registered into MNI space using non-linear registration with ANTs [33]. The calculation of a diffusion tensor using FSL tools yields FA maps and additional scalar images. Further quality control for FA maps was conducted to retain maps with a correlation greater than 0.9 with the mean FA map across all the individuals.

### 2.4. Development Changes in Gray Matter and White Matter

A mask was generated to include voxels with a mean value of GM and FA > 0.2 baseline samples separately and applied to each data. The same baseline mask was used for GM and FA’s two-year samples. The result obtained was a GM matrix of size 3302 × 490,539 for both the baseline and the two-year and FA matrix of size 3302× 94,440 for both the baseline and the two years. The change in the GM within two years, called ΔGM, was obtained by subtracting the baseline data from the two-year follow-up data. Similarly, the change in FA within two years, ΔFA, was identified. To reduce the data dimension, PCA was applied to each matrix of ΔGM and ΔFA to derive 100 features for each. The variance explained by the PCA features for GM and FA captures >80% and >65%, respectively. We sought to capture a large amount of variance while eliminating potential noise, and 100 GM principal components (PCs) captured >80% of total GM variance; however, to capture the same amount FA variance, 740 PCs were needed, which dramatically increased the chance of noise inclusion. We decided to include only the first 100 FA PCs that captured over 65% of the total variance, removing components each carrying <2% of variances. Thus, choosing 100 PCs for both image types allowed us to maximize relevant data representation, mitigate noise, and balance the input complexity from both image types.

### 2.5. CCA Feature Extraction

CCA is a statistical technique used to uncover linear relationships between two datasets [34]. Let *X* and *Y* denote two datasets, where $X\in {\mathbb{R}}^{n\times p}$ and $Y\in {\mathbb{R}}^{n\times q}$, with *n* as the number of samples and *p* and *q* as the numbers of variables for each dataset. The covariances of *X*, *Y*, and *XY* are represented by Σ_*X*_, Σ_*Y*_, and Σ_*XY*_, respectively.

CCA finds weight matrices *u* and *v* to maximize the correlation between the canonical covariates *Xu* and *Yv*. These weight matrices are linear projection vectors obtained by solving a generalized eigenvalue decomposition of a covariance matrix between the GM and FA datasets, ensuring that the transformed features are maximally correlated. The objective function for optimization is presented in Equation (1).

(1) $$max\;u^{T}\sum_{XY^{v}}=min\;{\left\|Xu-Yv\right\|}^{2}\;s.t.\;{\left\|Xu\right\|}_{2}={\left\|Yv\right\|}_{2}=1$$

### 2.6. DCCA Feature Extraction

Andrew et al. [26] first proposed the non-linear extension of CCA using neural networks called DCCA. The output of the neural networks is subjected to the computation of correlation scores. We used a more robust variation of DCCA named DCCAE [35], which combines the benefits of DCCA for capturing correlations and an autoencoder (AE) for learning better representations. The AE consists of an encoder that reduces input dimensionality and a decoder that reconstructs the data, minimizing the error. The reconstruction error for two datasets, *X* and *Y*, is calculated using the mean square error (MSE), as shown in Equation (2).

(2) $${\left(\text{Reconstruction}\;\text{Loss}\right)}_{X}=\underset{\theta_{1}}{min}\sum {\left\|X-\hat{X}\right\|}_{F}^{2}\;{\left(\text{Reconstruction}\;\text{Loss}\right)}_{Y}=\underset{\theta_{2}}{min}\sum {\left\|Y-\hat{Y}\right\|}_{F}^{2}$$

Here, *X* and *Y* represent original data, $\hat{X}$ and $\hat{Y}$ represent reconstructed data, and *θ*_1_ and *θ*_2_ are the network parameters of the decoders. For the two views, *X* and *Y*, consider two deep neural networks (DNNs) as encoders *f* and *g*, which extract non-linear features *f*(*X*) and *g*(*Y*), respectively. The latent representations are then subjected to CCA transformation, described by weights *u* and *v*, maximizing correlation, as shown in Equation (3). Thus, DCCAE first employs a nonlinear transformation with deep neural networks, learned by iteratively optimizing the cost function, and then it applies the CCA to the transformed latent data representation by computing *u* and *v* matrices. Altogether, the nonlinear transformation and *u* and *v* matrices define the latent DCCAE components.

(3) $$\begin{array}{c} \underset{u,v}{min}-\frac{1}{N}tr\left(uf(X)g{\left(Y\right)}^{T}v\right)+\\ \frac{\lambda }{N}\sum_{i=1}^{N}{\left\|X_{i}-p(f\left(X_{i}\right)\right\|}^{2}+{\left\|Y_{i}-q(g\left(Y_{i}\right)\right\|}^{2}\\ \;s.t.\;u^{T}\left(\frac{1}{N}f(X)f{\left(X\right)}^{T}+r_{x}I\right)u=I,\\ v^{T}\left(\frac{1}{N}g(Y)g{\left(Y\right)}^{T}+r_{y}I\right)v=I,\\ u_{i}^{T}f(X)g{\left(Y\right)}^{T}v_{j}=0,\;for\;i\neq j \end{array}$$

Here, *X*_*i*_ and *Y*_*i*_ are original data of two views, *p*(*f*(*X*_*i*_)) and *q*(*g*(*Y*_*i*_)) are reconstructed data of the two networks, *p* and *q* are decoder functions, *λ* is the weight parameter, and *r*_*x*_ and *r*_*y*_ are regularization parameters. DCCAE combines the dual objectives of correlation loss and reconstruction loss simultaneously. The model aims to minimize MSE for optimal learning representations and maximize the correlation between the two encoded sets.

We employed an open-source DCCAE model [35]. The model outputs correlation scores between GM and FA for a specified number of components. In the objective function, the weight value *λ* regulates both the reconstruction and correlation losses. We set a weight value of 0.08 for the reconstruction part and a weight value of (1 − 0.08) = 0.92 for the correlation part. The assignment of weight was determined through cross-validation scores.

### 2.7. Evaluation of Multimodal Latent Imaging Features

#### 2.7.1. CCA Implementation and Feature Interpretation

In our CCA analysis, we utilized 100 principal components (PCs) each for GM and FA. This choice of smaller variables (3302 × 100 for GM and 3302 × 100 for FA) than the total number of variables serves to mitigate the risk of overfitting. The dataset was partitioned into 80% for training and 20% for testing. We set the number of latent dimensions as 20 components, producing 20 correlation scores from the training samples. The derived CCA transformation was then applied to testing data to obtain 20 correlation scores. A correlation was deemed significant if the *p*-value of the correlation was less than 0.0025 in both the training and testing data, adhering to the Bonferroni correction of 20 tests.

For significantly correlated CCA components, we implemented the Linear Mixed Effect (LME) Model to find brain growth patterns’ association with cognition (fluid intelligence, crystallized intelligence, and the total composite score, separately) and behavioral changes. In the LME models, CCA-extracted components (GM and FA tested separately), age and sex are fixed-effect independent variables, the collection site is a random-effect variable, and a change in cognition or behavior is the dependent variable (tested separately for each measure). These analyses were conducted using hold-out testing data.

#### 2.7.2. DCCAE Implementation and Feature Interpretation

The same input data as in the CCA model were used in the DCCAE model. The partitioning of the dataset into training and testing samples mirrored that of the CCA model. During the training of the DCCAE model, we applied five-fold cross-validation to fine-tune hyperparameters, optimizing the sum of the first 10 correlation scores. The identified optimal hyperparameters included a batch size of 256,150 epochs, a learning rate of 0.01, and a neural network architecture (encoder) of a two-layer MLP with 100 and 20 neurons, respectively. The encoder’s output was utilized to extract canonical variables through the CCA method, with the latent dimensions set to 20. The best-performing DCCAE model was projected onto the testing sample, and, akin to the CCA approach, we determined the significance of components. These significant DCCAE components were evaluated using LME models to find the association between cognition and behavioral measures in a similar way through CCA.

To identify the significant contributing features, we applied the occlusion approach. Specifically, we set one input feature of GM or FA, separately, to zero while keeping all other features unchanged, applied the modified data to the model in order to obtain the latent representation, and computed the difference between the new latent representation and the original latent representation. Subsequently, the mean of each column of the resulting differences was calculated, forming a vector of size 1 × 20 (where 20 represents the latent dimensions). This process was repeated for each of the 100 GM or FA features, yielding a matrix of 100 × 20 for GM or FA, respectively, which we termed the contribution score matrix. From the contribution matrices, we calculated the absolute value and selected the top PC components for the DCCAE components.

#### 2.7.3. Relation Between CCA and DCCAE Components

To understand how the CCA latent representations are related to the DCCAE latent representation, we calculated the correlation between each of the significant components of CCA and each of the significant components of DCCAE. In addition, we tested the prediction power or explanation power of derived latent brain growth patterns for cognition and behavior. Specifically, for both the CCA and DCCAE components, we combined GM and FA components together as independent variables, along with age, sex, and site, to predict cognitive or behavioral changes using LME models. The total variance explained by the combined GM and FA components was computed for fluid intelligence, crystallized intelligence, and the total composite score and for each of the eight CBCL syndromes.

## 3. Results

### 3.1. Features Obtained from CCA Model

From the CCA model, we found 15 pairs (1st, 2nd, 3rd, 4th, 5th, 7th, 8th, 9th, 10th, 13th, 14th, 15th, 16th, and 19th) of GM and FA features to be significantly correlated (*p*-value < 0.0025) in the training samples, which was verified in the testing samples. The mean correlation scores in the training and testing of these 15 significant pairs were 0.34 and 0.21, respectively. Table 1 illustrates the correlation between the first five pairs of components and the respective *p*-value.

### 3.2. Features Obtained from DCCAE Model

From the DCCAE model, the first 13 pairs of latent GM and FA features were significantly correlated (*p*-value < 0.0025) and verified in testing samples. The mean correlation scores in the training, validation, and testing of these 13 significant pairs were 0.52, 0.35, and 0.33, respectively. Table 2 illustrates the correlation between the first five pairs of canonical variates and their respective *p*-value.

### 3.3. Correlation of CCA and DCCAE Components

We calculated the correlation between each of the 15 significant components of CCA and each of the 13 components of DCCAE. Out of the 15 components of CCA, 12 CCA GM components and 11 FA components showed a correlation >0.15 with at least one DCCAE component. As an example, Tables 3 and 4 present the correlation scores between the first three components of CCA with DCCAE components that have correlation scores greater than 0.15, along with their respective *p*-values.

### 3.4. Top Brain Regions Contributing to CCA and DCCAE Components

To illustrate the brain regions, the top PCs of the first three significantly correlated GM and FA components were plotted for both the CCA results and the DCCAE results. To plot the brain regions, the CCA/DCCAE components were traced back to PCA components using the weight matrix of CCA/DCCAE, and the top PCA components were then mapped to the brain regions.

**Figure 1.**
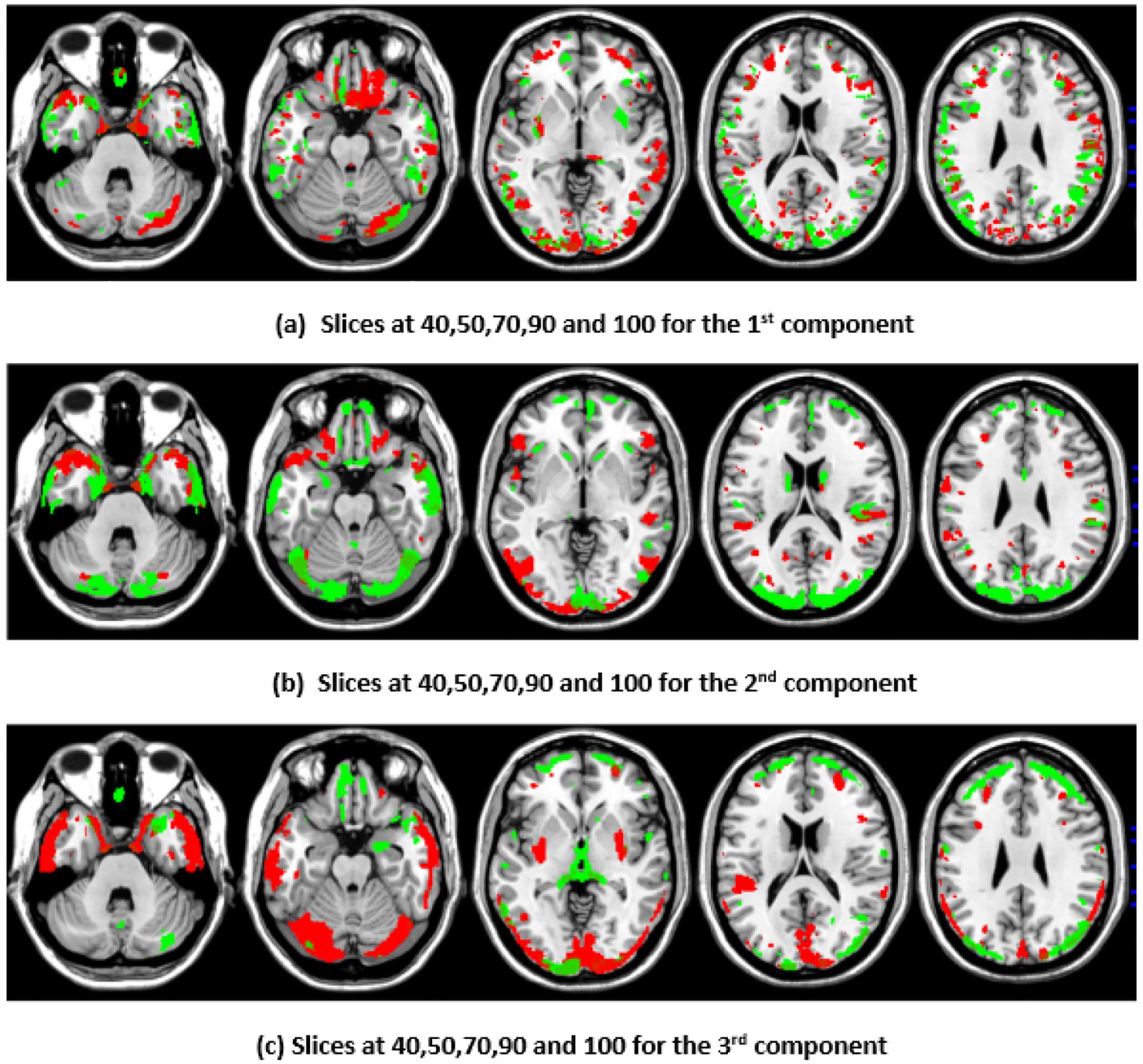
First, second, and third components of GM identified through CCA.

**Figure 2.**
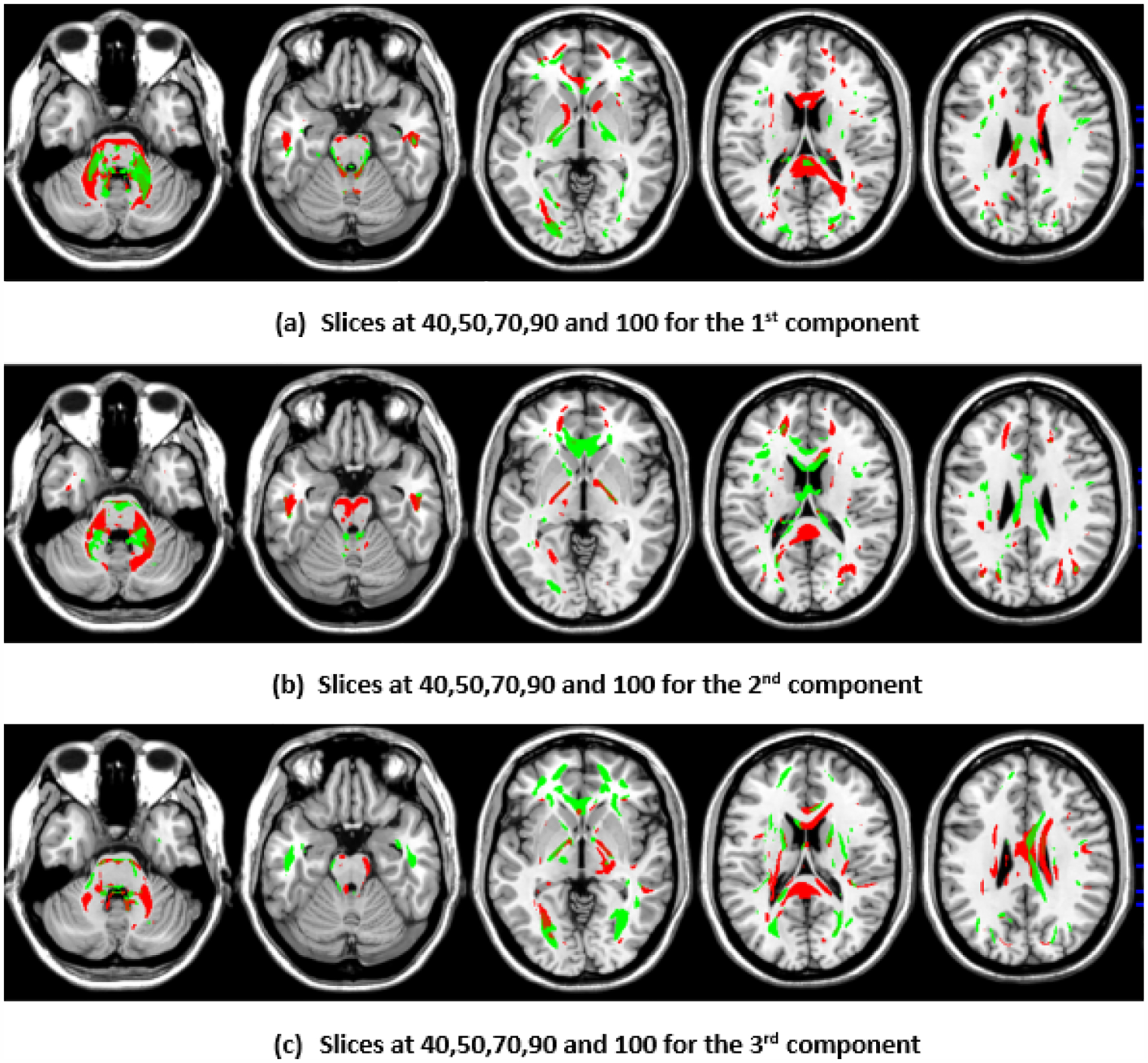
First, second, and third components of FA identified through CCA.

**Figure 3.**
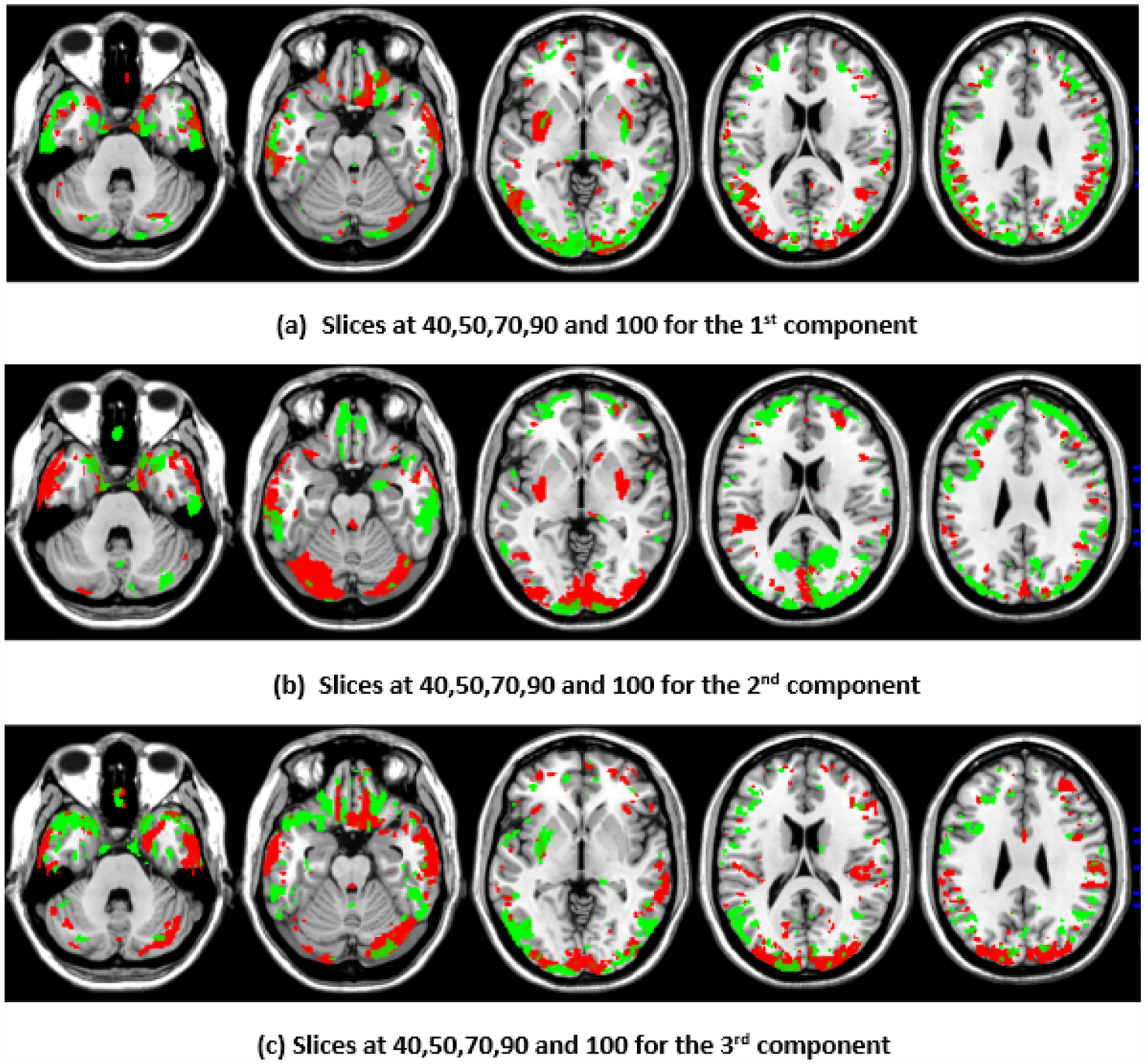
First, second, and third components of GM identified through DCCAE.

**Figure 4.**
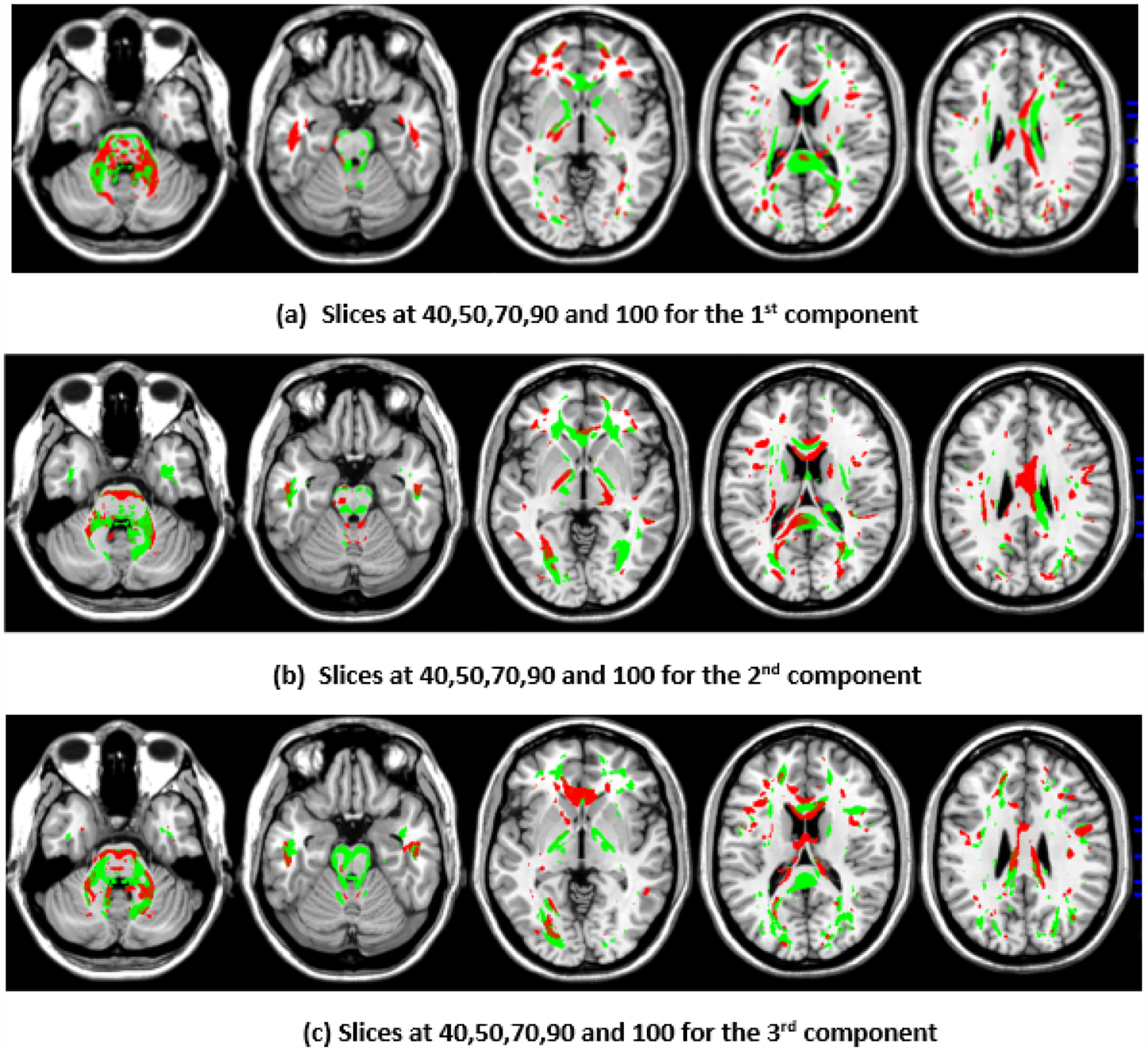
First, second, and third components of FA identified through DCCAE.

Figures 1 and 2 depict GM and FA regions for CCA, respectively, while Figures 3 and 4 portray GM and FA regions for DCCAE, respectively, with green indicating positively correlated GM and FA regions and red indicating negatively correlated regions. The GM regions identified via the first component of CCA with their respective volume in cubic centimeters (cc) include the middle temporal gyrus (12.1 cc), pre-central gyrus (14.5 cc), middle frontal gyrus (14.6 cc), superior frontal gyrus (2.0 cc), and sub-gyral regions (12.7 cc). The second component highlights the cuneus (27.3 cc), middle occipital gyrus (10.6 cc), superior frontal gyrus (6.3 cc), lingual gyrus (13.4 cc), and superior temporal gyrus (8.4 cc). The third component encompasses the superior frontal gyrus (27.2 cc), middle frontal gyrus (12.6), cuneus (23.6), superior temporal gyrus (10.8), middle occipitalgyrus (16.0 cc), and thalamus (17.4 cc). Similarly, the first component of DCCAE reveals Cuneus (15.4 cc), middle temporal gyrus (22.1 cc), precentral gyrus (15.6 cc), middle frontal gyrus (20.1 cc), superior temporal gyrus (6.8 cc), inferior parietal lobule (17.3 cc), and superior frontal gyrus (29.2 cc). The second component identifies cuneus (19.6 cc), middle occipital gyrus (11.1 cc), superior frontal gyrus (30.4 cc), middle frontal gyrus (20.1 cc), sub-gyral (9.1 cc), postcentral gyrus (9.5 cc), precuneus (6.0 cc), and middle temporal gyrus (10.3 cc). The third component includes the middle temporal gyrus (23.7 cc), precentral gyrus (11.0 cc), cuneus (29.0 cc), lingual gyrus (6.3 cc), middle occipital gyrus (14.3 cc), inferior frontal gyrus (2.3 cc), postcentral gyrus (10.8 cc), and inferior parietal lobule (6.8 cc).

Similarly, the FA regions identified via the first component of the CCA include the corticospinal tract (7.2 cc), anterior thalamic radiation (8.4 cc), and forceps minor (3.4 cc). The second component highlights the corticospinal tract (6.3 cc), anterior thalamic radiation (8.9 cc), forceps minor (9.8 cc), and inferior longitudinal fasciculus (1.2 cc). The third component recognizes anterior thalamic radiation (9.4 cc), the forceps minor (7.7 cc), the inferior longitudinal fasciculus (2.5 cc), and the inferior fronto-occipital fasciculus (2.7 cc). Likewise, the first component of DCCAE points out anterior thalamic radiation (13.1 cc), the forceps minor (7.9 cc), the inferior longitudinal fasciculus (3.4 cc), the corticospinal tract (13.0 cc), and the inferior fronto-occipital fasciculus (2.2 cc). The second component reveals the forceps minor (12.5 cc), the inferior longitudinal fasciculus (2.0 cc), the corticospinal tract (7.9 cc), and anterior thalamic radiation (12.7 cc). The third component includes the corticospinal tract (9,4 cc), anterior thalamic radiation (10.4 cc), and the forceps minor (11.0 cc).

### 3.5. Association of CCA and DCCAE Components with Cognition

From the LME models of CCA components and DCCAE components performed separately, Tables 5 and 6 show the GM and FA components that were associated with fluid intelligence, crystallized intelligence, and the total composite score. The computed *p*-value for each significant component indicates the strength of the association between the identified brain regions and cognition, while the sign denotes whether this association is positive or negative. The NA shows that none of the components were significant.

### 3.6. Association of CCA and DCCAE Components with CBCL

Tables 7 and 8 show the GM and WM components that were associated with CBCL’s syndrome scales from the LME models of CCA components and DCCAE components, respectively. The sign represents whether the association between the brain regions (identified via the mentioned components) and the CBCL is positive or negative. The NA shows that none of the components were significant.

### 3.7. Total Variation Explained by Brain Components of CCA and DCCAE

Table 9 shows the variance explained by CCA and DCCAE for cognition. Table 10 shows the variance explained by CCA and DCCAE for CBCL behavioral measures.

## 4. Ablation Study

We conducted an ablation study to examine the impact of non-linearity on the correlation strength in DCCAE. Specifically, our encoder model consists of one hidden layer with 100 neurons, which is the key element to model non-linearity. For the ablation study, we removed the hidden layer, resulting in an encoder composed solely of the input and output layers. We then assessed the correlation between the latent representations obtained from the output layers from the gray matter and white matter encoders. Our findings revealed that 16 pairs of GM and FA components were significantly correlated (*p*-value < 0.0025). The mean correlation scores for these 16 significant pairs were 0.52 in the training set, 0.44 in the validation set, and 0.38 in the testing set. Table 11 illustrates the correlations between the first five pairs of components. This ablation study demonstrates that the removal of the hidden layer resulted in a notable decrease in correlation scores, making them comparable to those obtained through linear CCA, as shown in Table I in the main text.

## 5. Discussion

In this study, we implemented both CCA and DCCAE models to extract correlated components from GM and FA changes within two years during adolescence, and such components represented brain growth patterns in GM density and FA integrity coherently in two years. Data from over three thousand children from the general population were used, and similar numbers of GM-FA component pairs were extracted and verified from the CCA and DCCAE models. A comparison of correlation strength suggests that the DCCAE yielded highly correlated GM–FA pairs in both training and testing data when compared to the CCA results. In both CCA and DCCAE, the correlation scores in the testing data were lower than those in the training data; this attenuation was expected when applying the model to independent testing data. Importantly, the persistence of statistically significant correlations in the testing data demonstrates the robustness of the model and suggests that the observed relationships are not a result of overfitting but, rather, reflect genuine associations between GM and FA features. CCA, being a linear model, only captures linear interactions among brain regions and thus a linear relationship between the GM and FA of white matter. On the other hand, DCCAE incorporates a deep learning architecture that is able to extract both linear and non-linear interactions among brain regions and thus a more intricate relation between the GM and FA of white matter. We speculate that this may be the main reason for stronger relations between GM and FA matter components in the DCCAE results.

When examining the direct similarity between components of CCA and DCCAE, we found that most of the CCA components (for both GM and FA) have high and significant correlations (shown in Tables 3 and 4) with some of the DCCAE components. For example, CCA GM component first is significantly and negatively correlated to DCCAE GM components first and second, and it is positively correlated to DCCAE GM component third. Similar results were observed for FA components; CCA FA component first was linked to DCCAE FA components first and second negatively and component third positively. The negative correlation observed in Tables 3 and 4 does not indicate a fundamental contradiction in the relationship between GM and FA features. Instead, it suggests that, for the same brain region, CCA encodes a change in one direction (an increase or a decrease), while DCCAE encodes it in the opposite direction. However, the underlying GM-FA association remains consistent across both models. This difference in sign can be further illustrated in Figures 1 and 3, where the first components from CCA and DCCAE show similar spatial patterns, particularly in the posterior occipital region, but the color representation differs, indicating an increase in one model and a decrease in the other.

We also examined the contributing brain regions of components that shared the same brain regions. The top PCs of the CCA and DCCAE components highlighted common regions for GM and FA. The first CCA component correlates with the first, second, and third DCCAE components. The brain regions of the first CCA components include the middle temporal gyrus, precentral gyrus, middle frontal gyrus, superior temporal gyrus, and sub-gyral while DCCAE’s first component identified the middle temporal gyrus, middle frontal gyrus, and sub-gyrus, DCCAE’s second component identified middle temporal gyrus, and middle frontal gyrus, and DCCAE’s third component identified the superior temporal gyrus and precentral gyrus. Similarly, common FA regions were also observed for CCA FA components and DCCAE FA components. The brain regions of the first CCA components include the corticospinal tract, anterior thalamic radiation, and the forceps minor, and DCCAE’s first, second, and third components identified brain regions pointed out by the first component of CCA.

The significant correlations between CCA and DCCAE components and the shared brain regions of components from the two approaches suggest that both linear and nonlinear approaches extracted similar brain growth patterns in the two years. Maybe two years is a short enough time for non-linear growth patterns in the brain structure to be estimated in a linear fashion with reasonable accuracy.

The GM brain regions identified using CCA and DCCAE show a negative association with cognition, including the superior frontal gyrus, middle temporal gyrus, and precuneus, while a positive association is observed with cognition in regions such as the inferior frontal gyrus and medial frontal gyrus identified through CCA. However, for the FA, only CCA components are related to fluid intelligence, and the brain regions are negatively related, which includes anterior thalamic radiation and the forceps minor.

Regarding the CBCL behavioral syndromes, five CCA GM components showed significant associations with aggressive behavior, somatic complaints, withdrawn/depressed, and thought problems. These components include brain regions such as the superior temporal gyrus, middle temporal gyrus, inferior frontal gyrus, and middle frontal gyrus and cuneus, which are negatively related to behavior. For white matter, CCA showed associations for the anxious/depressed, attention problem, and withdrawn/depressed conditions, which include negatively related brain regions such as the superior longitudinal fasciculus, anterior thalamic radiation, and the corticospinal tract, while thought problems had positive associations with the brain region of the inferior longitudinal fasciculus and forceps minor.

Similarly, the CBCL behavioral syndromes analysis using DCCAE with gray matter revealed negative associations for the aggressive behavior, anxious/depressed, and somatic complaints conditions, including the brain regions of the superior temporal gyrus, inferior frontal gyrus, superior frontal gyrus, middle temporal gyrus, middle frontal gyrus, cingulate gyrus, sub-gyrus, and precentral gyrus, along with positive associations for attention problems and withdrawn/depressed behavior with the brain regions of the inferior frontal gyrus and superior temporal gyrus. In white matter, DCCAE showed that the brain region has negative associations for attention problems, rule-breaking behavior, and somatic complaints, indicating the following regions: anterior thalamic radiation, the corticospinal tract, the forceps minor, and the inferior fronto-occipital fasciculus. Meanwhile, thought problems had positive associations with the brain regions of the forceps minor and the inferior longitudinal fasciculus.

Many studies have documented diverse growth patterns for different brain regions [36–39]. Particularly, the GM densities of brain regions presenting inverted U-shaped growth trajectories peaked at different times [40]. Our findings indicate that, between the ages of 10 and 12, children’s cognitive ability improves, accompanied by their behavioral changes, and such changes might be underscored by a GM density reduction in the negatively associated brain regions, a pruning phase of brain maturation, and also a GM density increase in the positively associated brain regions that are still in a growth phase of brain maturation.

The examination of the total variance in cognitive and behavioral changes explained by the components extracted from CCA or DCCAE indicates that brain growth patterns could explain cognitive maturation much better than behavioral changes, and the CCA and DCCAE components overall demonstrate a comparable ability in terms of explaining cognitive and behavioral changes.

## 6. Conclusions

We know that DCCAE is a more complex model compared to CCA and that it can learn non-linear patterns in data. However, this complexity also increases the risk of overfitting and has a high chance of capturing noise as well. Even though the DCCAE exceeds the CCA in terms of the correlation between GM–FA components in both the training and testing datasets, there is no increase in the number of meaningful components that are associated with cognition and behavior. Since CCA and DCCAE extract components that identify similar brain regions associated with both cognition and CBCL, and the variances explained by CCA and DCCAE brain components for cognition and behavior are approximately the same, taking into account the extra computational cost and complexity of DCCAE, we can use the simple CCA method for extracting latent features. So, when comparing the linear and non-linear approaches to capturing the brain growth pattern within two years, we found that the linear approach of CCA can provide a good estimation of changes, even though a literature review shows that brain growth follows a non-linear pattern.

In our study, behavioral and cognitive measures were analyzed separately to examine their association with brain structural changes in brain development. Specifically, cognitive measures (i.e., fluid intelligence, crystallized intelligence, and the total composite score) were investigated independently from eight CBCL behavioral syndromes. This approach allows us to identify neurobiological contributions to cognitive function or behavioral regulation, providing a clearer understanding of how brain structure supports these separate domains. While the interesting relationship between cognition and behavior, as well as the potential modulation or mediating relation between the brain, cognition, and behavior, is important and worth an in-depth investigation, in our current results, GM CCA components 1, 2, and 7, the 9th component of GM DCCAE, and the 14th component of FA CCA are associated with both cognition and behavior. These results suggest that the same brain regional growth patterns may support cognitive maturation and behavior change during development. This relationship warrants further investigation.

The findings of this study should be interpreted with a consideration of the following limitations. We used the linear approach of PCA to reduce the dimension of GM and FA to 100 features, and we implemented the non-linear method of DCCAE to extract the correlated features. So, the entire comparison is constrained to PCA input features. Future investigations of fully non-linear approaches such as the use of a convolutional neural network to directly extract the hidden features from 3D images are necessary. Brain structural changes beyond two years may be more suitable for testing non-linear versus linear growth patterns, given that brain growth patterns within two years are linear.

## Figures and Tables

**Table 1.** Correlation of CCA features in training and testing.

Score	1st Comp	2nd Comp	3rd Comp	4th Comp	5th Comp
Training	0.61 (1.8 × 10^−178^)	0.54 (4.2 × 10^−120^)	0.48 (7.8 × 10^−114^)	0.33 (5.8 × 10^−48^)	0.32 (2.5 × 10^−40^)
Testing	0.22 (7.7 × 10^−09^)	0.44 (3.6 × 10^−33^)	0.32 (2.8 × 10^−17^)	0.12 (1.2 × 10^−04^)	0.26 (1.7 × 10^−12^)

**Table 2.** Correlation of DCCAE features in training, validation, and testing.

Score	1st Comp	2nd Comp	3rd Comp	4th Comp	5th Comp
Training	0.82 (4.1 × 10^−293^)	0.74 (3.5 × 10^−250^)	0.71 (2.1 × 10^230^)	0.68 (1.2 × 10^−210^)	0.60 (5.3 × 10^−160^)
Validation	0.61 (2.4 × 10^−55^)	0.54 (6.8 × 10^−41^)	0.51 (2.2 × 10^−35^)	0.45 (6.1 × 10^−26^)	0.41 (3.5 × 10^−20^)
Testing	0.58 (4.7 × 10^−50^)	0.54 (6.7 × 10^−41^)	0.46 (1.3 × 10^−28^)	0.40 (5.2 × 10^−19^)	0.38 (7.8 × 10^−17^)

**Table 3.** Correlation and *p*-value of CCA and DCCAE latent features (gray matter).

CCA Components	DCCAE Components	Correlation Score	*p*-Value
1st	1st	−0.46	3.10 × 10^−174^
	2nd	−0.20	6.3 × 10^−32^
	3rd	0.32	2.34 × 10^−84^
2nd	1st	−0.18	3.83 × 10^−27^
	2nd	−0.19	1.97 × 10^−30^
	3rd	−0.41	4.33 × 10^−139^
3rd	1st	−0.30	1.13 × 10^−71^
	2nd	0.38	5.32 × 10^−119^
	5th	−0.20	1.50 × 10^−32^

**Table 4.** Correlation and *p*-value of CCA and DCCAE latent features (white matter).

CCA Components	DCCAE Components	Correlation Score	*p*-Value
1st	1st	−0.43	3.38 × 10^−149^
	2nd	−0.22	1.20 × 10^−37^
	3rd	0.33	1.20 × 10^−85^
2nd	1st	−0.23	1.91 × 10^−43^
	2nd	−0.24	6.67 × 10^−46^
	3rd	−0.44	4.44 × 10^−161^
	5th	−0.24	2.48 × 10^−47^
3rd	1st	−0.48	3.02 × 10^−194^
	2nd	0.46	1.90 × 10^−174^

**Table 5.** Association of CCA’s GM and FA components with cognition.

	Gray Matter (CCA Components)	White Matter (CCA Components)
Fluid	2nd comp (0.03) (−), 7th comp (0.01) (−)	13th comp (0.04) (−), 14th comp (0.04) (−)
Crystallized	9th comp (0.03) (−)	NA
Total composite	1st comp (0.04) (+), 10th comp (0.04) (−)	NA

**Table 6.** Association of DCCAE’s GM and FA components with cognition.

	Gray Matter (DCCAE Components)	White Matter (DCCAE Components)
Fluid	NA	NA
Crystallized	9th comp (0.03) (−)	NA
Total composite	1st comp (0.04) (−), 9th comp (0.04) (−)	NA

**Table 7.** Association of CCA components with CBCL syndrome.

	Gray Matter (CCA Components)	White Matter (CCA Components)
Aggressive behavior	3rd comp (0.03) (−), 4th comp (0.04) (−)	NA
Anxious/depressed	NA	14th comp (0.023) (−)
Attention problems	NA	4th comp (0.02) (−)
Rule-breaking behavior	NA	NA
Somatic complaints	1st comp (0.01) (−), 7th comp (0.03) (−)	NA
Social problems	NA	NA
Thought problems	2nd comp (0.02) (−)	5th comp (0.02) (+), 15th comp (0.01) (+)
Withdrawn/depressed	2nd comp (0.03) (−), 3rd comp (0.01) (−)	7th comp (0.03) (−)

**Table 8.** Association of DCCAE components with CBCL syndrome.

	Gray Matter (DCCAE Components)	White Matter (DCCAE Components)
Aggressive behavior	5th comp (0.01) (−), 8th comp (0.01) (−)	NA
Anxious/depressed	5th comp (0.03) (−)	NA
Attention problems	9th comp (0.01) (+)	7th comp (0.03) (−)
Rule-breaking behavior	NA	4th comp (0.01) (−)
Somatic complaints	4th comp (0.04) (−), 9th comp (0.01) (−)	5th comp (0.03) (−)
Social problems	NA	NA
Thought problems	NA	2nd comp (0.04) (+)
Withdrawn/depressed	3rd comp (0.04) (+)	NA

**Table 9.** Total variance explained by CCA and DCCAE components for cognition.

	Variance Explained by	
	CCA components (%)	DCCAE components (%)
Fluid	11.11	9.8
Crystallized	9.99	3.09
Total composite	14.0	16.27

**Table 10.** Total variance explained by CCA and DCCAE components for CBCL.

	Variance Explained by	
	CCA components (%)	DCCAE components (%)
Aggressive behavior	1.01	0.7
Anxious/depressed	1.03	1.01
Attention problems	2.04	1.5
Rule-breaking behavior	1.01	1.23
Somatic complaints	1.11	1.01
Social problems	1.28	2.04
Thought problems	3.09	1.01
Withdrawn/depressed	2.04	2.04

**Table 11.** Correlation of DCCAE features in training, validation, and testing without hidden layers.

Score	1st Comp	2nd Comp	3rd Comp	4th Comp	5th Comp
Training	0.63	0.57	0.52	0.46	0.43
Validation	0.60	0.49	0.40	0.37	0.35
Testing	0.49	0.43	0.36	0.34	0.30

## Data Availability

Data will be made available upon request.
